# Theory of Mind Performance Predicts tDCS-Mediated Effects on the Medial Prefrontal Cortex: A Pilot Study to Investigate the Role of Sex and Age

**DOI:** 10.3390/brainsci10050257

**Published:** 2020-04-28

**Authors:** Maria Cotelli, Rosa Manenti, Elena Gobbi, Ivan Enrici, Danila Rusich, Clarissa Ferrari, Mauro Adenzato

**Affiliations:** 1 Neuropsychology Unit, IRCCS Istituto Centro San Giovanni di Dio Fatebenefratelli, Via Pilastroni, 4, 25125 Brescia, BS, Italy; 2 Department of Philosophy and Educational Sciences, University of Turin, 10124 Turin, TO, Italy; 3 Department of Human Science, LUMSA University (Libera Università Maria Santissima Assunta), 00193 Rome, RM, Italy; 4 Statistics Service, IRCCS Istituto Centro San Giovanni di Dio Fatebenefratelli, 25125 Brescia, BS, Italy; 5 Department of Psychology, University of Turin, 10124 Turin, TO, Italy

**Keywords:** theory of mind, transcranial Direct Current Stimulation (tDCS), sex differences, noninvasive brain stimulation, social cognition, aging

## Abstract

Transcranial Direct Current Stimulation (tDCS) has become an increasingly promising tool for understanding the relationship between brain and behavior. The purpose of this study was to investigate whether the magnitude of sex- and age-related tDCS effects previously found in the medial prefrontal cortex (mPFC) during a Theory of Mind (ToM) task correlates with social cognition performance; in particular, we explored whether different patterns of activity would be detected in high- and low-performing participants. For this, young and elderly, male and female participants were categorized as a low- or high-performer according to their score on the Reading the Mind in the Eyes task. Furthermore, we explored whether sex- and age-related effects associated with active tDCS on the mPFC were related to cognitive functioning. We observed the following results: (i) elderly participants experience a significant decline in ToM performance compared to young participants; (ii) low-performing elderly females report slowing of reaction time when anodal tDCS is applied over the mPFC during a ToM task; and (iii) low-performing elderly females are characterized by lower scores in executive control functions, verbal fluency and verbal short-term memory. The relationship between tDCS results and cognitive functioning is discussed in light of the neuroscientific literature on sex- and age-related differences.

## 1. Introduction

Aging is accompanied by changes in cognitive abilities and some of these abilities are more vulnerable to aging than others. The impact of aging on social cognition skills is an important question. Theory of Mind (ToM) is the ability to predict other people’s actions and to explain them in terms of their underlying beliefs, intentions or feelings [1,2]. Furthermore, ToM is the core of social interaction and difficulties in these abilities have been reported in several neuropsychiatric disorders [3,4,5,6]. It has been suggested that ToM performance is based on a distributed neural network involving the temporo-parietal junctions (TPJs), the precuneus and the medial prefrontal cortex (mPFC), with a pivotal role assigned to this latter brain area [7,8].

The findings of previous studies have been somewhat inconsistent in relation to the changes in ToM performance during physiological aging [9]. Sullivan and Ruffman [10] have shown that a proportion (13%–33%) of elderly adults exhibit unimpaired performance on ToM tasks. However, a recent literature review about physiological and pathological aging concluded that a normal aging-related decline is evident in social cognition abilities [9].

Another important question about ToM relates to the influence of sex differences on social cognitive processes [11]. According to Baron-Cohen’s model [12], females are, on average, more inclined toward activities that require an empathizing style; conversely, males are, on average, more skilled at “systemizing” than females. Accordingly, behavioral studies indicate that female participants perform better than male individuals on emotion recognition [13], social sensitivity [14], empathy [15] and emotional intelligence [16] tasks.

Regarding healthy aging, some neuroimaging studies have shown age-related structural and functional changes that could be linked to variability in ToM task performance. In particular, white matter connectivity and loss of integrity have been reported, as well as increased activity in the inferior frontal cortex (IFC) and decreased activity in the medial frontal cortex [17,18]. Determining whether these observed differences are attributable to a compensatory mechanism or whether they are indicative of aging-related impairments in ToM performance, is challenging.

In addition to neuroimaging, recent years have seen the use of transcranial Direct Current Stimulation (tDCS), a well-tolerated neuromodulation technique [19], to study social cognition abilities [20]. Based on polarity (anodal or cathodal) and on the initial neural activation state of the stimulated regions, tDCS can increase or decrease cortical excitability, although polarity-specific effects are not clear-cut and cathodal stimulation often results in weaker effects [21].

In the neuroscience literature, tDCS has become an increasingly promising tool for understanding the relationship between brain and behavior both in healthy subjects and in clinical populations [22]. Several studies have suggested that this technique can modulate functions in healthy aging and in several neurological and psychiatric disorders [23,24]. To date, only a few studies have assessed the effects of tDCS on social cognition. A recent review [20] has suggested that tDCS can modulate social abilities, suggesting that this methodology may represent a highly valuable tool for deepening our understanding about the neural basis of social abilities.

Regarding sex-related differences in social cognition, tDCS studies have rarely explored this issue [25,26]. In a previous tDCS study conducted by our group in a sample of young participants, we found evidence of sex-related differences in performance on the Attribution of Intentions (AI) task, a ToM task assessing the ability to represent other people’s intentions from the observation of their daily actions. In particular, we identified a significant interaction between sex and tDCS condition (active vs. sham applied over the mPFC), with improved performance during active tDCS (with the anode placed over the mPFC and the cathode located between Oz and Inion) in females only [27]. Moreover, in a recent study, Martin and collaborators [28] showed improved performance in a ToM task, Reading the Mind in the Eyes (RME), after the application of high-definition transcranial Direct Current Stimulation (HD-tDCS) to the dorsomedial frontal cortex in young females only. Recently, we conducted a double-blind study, applying tDCS on the mPFC (anodal, cathodal and sham tDCS) to modulate elderly participants’ performance on the AI task [29]. In line with previous studies on young individuals, indicating that females have a greater sensitivity to social stimuli, we also found sex-related differences in association with active tDCS (with the anode placed over the mPFC and the cathode located between Oz and Inion) on ToM performance in a group of healthy elderly participants [29]. In particular, a single session of anodal tDCS over the mPFC led to a significant slowing of responses in the AI task relative to sham in a sample of aged females. No effects were found in elderly male individuals or in the cathodal stimulation group.

It remains unclear whether these different tDCS sex-related effects in young and in elderly individuals may be beneficial to maintain social cognition in healthy aging. To address this issue, we carried out this pilot study, which involved re-analyzing data acquired in our previous studies [27,29]. The purpose of this pilot study was to investigate whether the magnitude of the sex- and age-related tDCS effects during a ToM task correlated with social cognition performance, suggesting the presence of different effectiveness windows for tDCS among high- and low-performing participants. We took this approach to take advantage of the ability of tDCS to modify cortical excitability and to test its neuromodulatory effects. We applied tDCS, an effective, safe and low-cost tool, to modulate ongoing brain activity, to establish the causality in the brain–behavior relationship and to further explore the slowing of ToM responses during anodal tDCS over the mPFC relative to sham, which was previously recorded exclusively in elderly females [29]. Based on our previous studies among young [27] and elderly [29] participants on the different effects of tDCS according to sex in both young and elderly participants, we conducted a pilot study investigating the possible influence of age and sex on the magnitude of the tDCS effect.

To address this question, elderly and young participants were categorized as low- or high-performers on the basis of their RME task score [30]. RME is the most widely used task in adults to detect individual differences in ToM performance. While the AI task is designed to capture ToM differences between groups in terms of reaction times (and was developed in the context of neuroimaging and brain stimulation studies [31,32,33,34,35,36], the RME is designed to capture ToM differences in terms of accuracy in behavioral performance. Nevertheless, neuroimaging meta-analyses [8,37] have shown that the mPFC emerges across ToM experimental paradigms as a key area of activation regardless of task-specific demands. Thye et al. [38], using an independent component analysis, specifically compared RME with a causality task involving intentional attribution (conceptually identical to the AI task), identifying the mPFC in the top-ranked components, confirming the key role of this brain region in general ToM processing, regardless of the task type. Thus, based on this evidence, we used RME to categorize participants in terms of ToM performance—and the AI task to test tDCS effects on these abilities.

Finally, we explore the relationship between active tDCS effects and the individual performance in ToM tasks, executive functions, language abilities and memory capacities.

On the basis of existing literature indicating (i) sex-related differences in ToM performance in both young and elderly individuals, (ii) the role of mPFC in ToM performance and (iii) slowing of reaction times (RTs) induced by a single session of anodal tDCS over mPFC, compared to sham in an elderly sample, we expected to find sex- and age-related differences in the magnitude of the effects on ToM performance induced by anodal tDCS over mPFC.

## 2. Materials and Methods

### 2.1. Participants

In the present work, we reanalyzed the data acquired in two previous studies [27,29]. As we aimed to broaden the effects of anodal tDCS over mPFC relative to sham, we included, in the present analysis, young and elderly individuals who underwent anodal vs. sham tDCS over the mPFC in our previous studies: 32 healthy young volunteers (16 females and 16 males; mean age: 23.6; SD 3.6 years) [27] and 30 healthy elderly participants (15 females and 15 males; mean age: 67.9; SD 6.1 years) [29]. These two groups were included in two different studies with the same experimental design and procedures apart from the intensity of the tDCS administered.

Exclusion criteria were as follows: (a) history of traumatic brain injury, brain tumor or stroke; (b) prior or current neurological or major psychiatric disorders; (c) history of alcohol abuse; (d) any contraindication to tDCS. All elderly participants underwent detailed neuropsychological evaluation and a pathological score in one or more of the neuropsychological tests represented further exclusion criteria for elderly participants (see Supplementary Table S1 for details).

All participants were explained about the aims of the research and written informed consent was obtained. The study was approved by the ethics committee of the Istituto di Ricovero e Cura a Carattere Scientifico (IRCCS) Centro San Giovanni di Dio, Fatebenefratelli, Brescia, Italy (n. 41/2016) and was conducted in accordance with the Declaration of Helsinki.

### 2.2. Procedure

#### 2.2.1. Clinical and Neuropsychological Assessment

All elderly participants underwent extensive clinical and neuropsychological evaluations carried out over two sessions. The cognitive assessment included test for evaluation of global cognitive abilities, such as the Mini Mental State Examination (MMSE) [39] and tests for verbal fluency (phonemic and semantic), language comprehension (Token Test), object and action naming (BADA), word knowledge (Wechsler Adult Intelligence Scale-Vocabulary subtest), as well as non-verbal reasoning (Raven’s Colored Progressive Matrices), visuo-spatial capacity (Rey-Osterrieth Complex Figure: Copy), attention and executive functions (Trail Making Test, Stroop Test, Wisconsin Card Sorting Test, Flanker Task) and memory (Rey Auditory Verbal Learning Test, immediate and delayed recall, Story Recall, Rey-Osterrieth Complex Figure Recall and Digit Span Forward and Backward). All tests were administered and scored according to standard procedures [40].

The 30-item version of the Geriatric Depression Scale (GDS) [41] and the State-Trait Anxiety Inventory (STAI) [42] were administered to exclude symptoms of depression and anxiety. The Interpersonal Reactivity Index (IRI) was included as a measure of empathy [43]. To obtain a Subjective Memory Complaints measure, the 28-item version of the Everyday Memory Questionnaire (EMQ) was used for the evaluation of memory complaints [44]. In addition, we administered the Cognitive Reserve Index (CRI) questionnaire, which provides a standardized measure of the cognitive reserve accumulated by individuals throughout their lifespan [45].

#### 2.2.2. Theory of Mind Assessment

All participants performed two ToM tasks: the RME task without the application of tDCS, to assess individual ToM performance and the AI task during active or sham tDCS, to investigate the effects of active tDCS on ToM performance.

##### 2.2.2.1. Reading the Mind in the Eyes Task

The RME task was performed before the beginning of the first tDCS session. The RME is an advanced ToM task evaluating the participant’s ability to represent others’ mental states by observing only their eyes [30]. Thirty-six black-and-white photographs of the eye regions of different males and females combined with four words that describe mental states are shown to participants. Participants are required to choose which of the four words printed around the pictures best describes what the person in the photograph is thinking or feeling. There is only one correct answer. The total number of correct choices (range: 1–36) represents the RME task score.

##### 2.2.2.2. Attribution of Intentions Task

We tested the effects of active tDCS (vs. sham tDCS) on ToM performance using an AI task. Our AI task is a video version of the ToM task used previously in healthy individuals and in people with neurological diseases [27,31,32]. Videos on short stories lasting 1500 ms were displayed on a computer screen, followed by two images representing two possible story endings. Participants were asked to choose the appropriate story ending, pressing the right or left button on the button box. This was done to demonstrate their comprehension of the displayed videos stories. Only one of the pictures constituted the correct conclusion of the story. The visual location of the correct answer (right or left) was randomized and the two possible story endings were shown until a response was recorded. Participants were seated in a quiet room in front of a computer monitor, and the task was displayed using Presentation software [46]. Accuracy (percentage of correct responses) and RTs (time from the picture appearing until the answer) were recorded.

**Figure 1:**
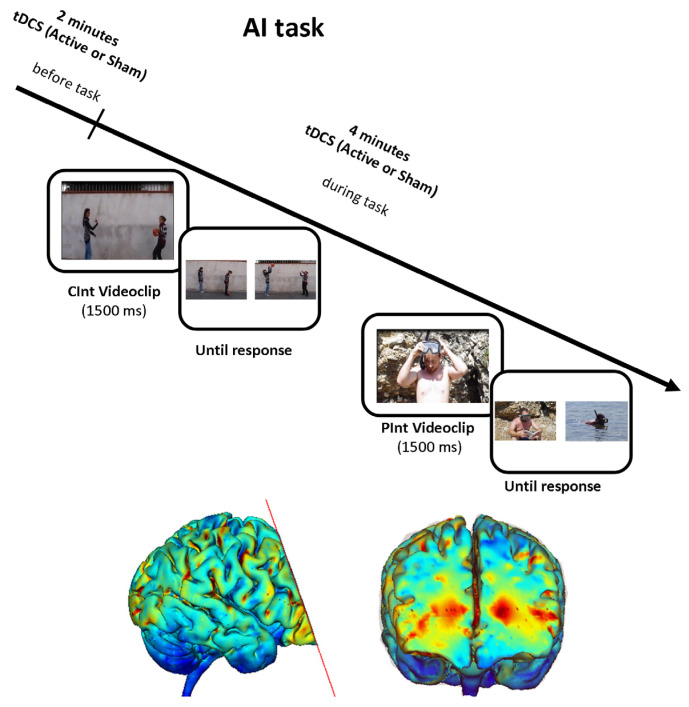
Experimental design and current flow model for transcranial Direct Current Stimulation (tDCS) application. In the Attribution of Intentions (AI) task, a short video was played, and the participant was asked to choose the picture representing a logical story ending by pushing one of the two buttons on the button box. One example for each stimulus condition (Communicative Intention (CInt) and Private Intention (PInt)) is displayed. Active or sham tDCS was started two minutes before the beginning of the experimental task and continued throughout the AI task. We utilized two 7 cm × 5 cm sponge pads and the current flow model is represented in the transverse view and 3D view on Male 1 model in Soterix HD Targets software (Soterix Medical, New York, NY, USA). The Soterix HD Targets software program is a simple tool to visualize current flow through the head, determined by the selected montage. For current flow calculation, we selected a uniform distribution over all electrodes because it leads to the most robust results, with an error of no more than 6%.

Two experimental conditions were used: (a) the Private Intention condition (PInt), in which the actions of one individual were shown in the stories; (b) the Communicative Intention condition (CInt), in which videos displayed the social interaction. In total, 34 CInt stories and 34 PInt stories were used in our study, split into two mixed blocks of 34 stimuli (17 PInt and 17 CInt stimuli) each. Each mixed block of stories corresponded to one of the two stimulation types (active and sham stimulation). Figure 1 shows a graphical representation of the task.

Both the stimulation conditions (active and sham tDCS) and the presentation order of the two mixed story blocks were randomized across participants. The two tDCS sessions were administered on two consecutive days at the same time of the day to minimize confounding factors.

#### 2.2.3. tDCS Procedure

tDCS was applied using a battery-driven constant-current stimulator (BrainStim, EMS; Bologna, Italy) through a pair of rubber electrodes covered by saline-soaked sponges (7 cm × 5 cm). The selected target area was mPFC, as it was identified as a pivotal brain area for ToM performance in our previous functional Magnetic Resonance Imaging (fMRI) studies [33,34,35,36]. The anode was placed over the mPFC (i.e., Fpz site) and the cathode was positioned between Oz and Inion as previously reported [31,32]. This montage was selected because extracephalic montages could create current densities in deep brain regions, especially in the white matter [47]. Therefore, we used a current flow model analysis (Soterix Medical, New York, NY, USA) for selecting an appropriate montage of commonly used electrode configurations (Figure 1). During active tDCS, a constant current of 1 mA for young participants and 1.5 mA for elderly participants was applied for 6 minutes (with a ramping period of 10 seconds at the beginning of the stimulation), starting 2 minutes before the beginning of AI task and finishing after the end of the task. The current density (young group: 0.029 mA/cm^2^; elderly group: 0.043 mA/cm^2^) was maintained below the safety limits [48].

These parameters are in line with those reported in most published tDCS studies, wherein electrodes are typically sized between 25 and 35 cm^2^ and a current of 1–2 mA is applied for a duration ranging between 5 and 30 min [23,49,50,51,52,53,54,55]. It is generally assumed that a minimum current density threshold of 0.017 mA/cm^2^ is necessary to actively modulate cortical activity [52]. Several studies have shown that a tDCS current density between 0.029 mA/cm^2^ and 0.08 mA/cm^2^ is sufficient to modify cognitive processes such as memory and learning, executive functions, language, perception and social cognition [20,51,55].

Modeling studies have demonstrated that, even when the electrode montage is kept consistent, the distribution of the current flow induced by tDCS and the effectiveness of stimulation can vary across subjects due to anatomic features such as skull thickness and composition or presence of lesions [56]. An important brain area associated with continuous changes over the lifetime is the prefrontal cortex, which, in elderly adults, is characterized by reduced cortical thickness and brain volume [57]. Since variations in frontal cortical architecture have been demonstrated as associated with reliable differences in the effects of tDCS [58], the effects of tDCS on this area can be stronger in younger participants than in the elderly and, consequently, we decided to use a higher current intensity in the elderly. In the sham stimulation condition, the tDCS procedure was the same, but the current was turned off 10 s after the beginning of the stimulation and turned on for the last 10 s, making this condition indistinguishable from the active stimulation. Active and sham tDCS were delivered using numeric codes, allowing for blinding of the researcher that administered the tDCS session.

At the end of each tDCS session, we asked the participants to complete a questionnaire on the perceptual sensations they experienced during tDCS to detect sensation differences during active and sham sessions and to identify potential side effects of the stimulation.

#### 2.2.4. Statistical Analyses

Statistical analyses were performed using Statistica software version 10 (Dell Software, Aliso Viejo, CA, USA). Statistical significance was set at *p* < 0.05.

In the main analysis, we included active tDCS effects data (active minus sham RTs), representing the magnitude of the tDCS effects, to investigate whether the magnitude of the sex- and age-related tDCS effects during a ToM task correlated with RME task performance. Accordingly, the variable ‘performance’ (with high and low categories) was defined based on the median value of the RME task in each of the four groups: young female participants, young male participants, elderly female participants, elderly male participants. Subsequently, an analysis of variance (ANOVA) was carried out to evaluate for differences between the groups: a 4 (Group: young female participants group, young male participants group, elderly female participants group, elderly male participants group) × 2 (Performance: high or low) ANOVA was performed.

To test for possible differences between the groups in the RME and AI tasks in the experimental condition without active tDCS, ANOVA was performed on the RME task score and the AI sham performance (accuracy and RTs). Post hoc analysis using Bonferroni corrections for multiple comparisons was used. 

Elderly female participants and elderly male participants were split into high- and low- performing subgroups and t-tests were carried out to evaluate for differences in any clinical or neuropsychological data between high and low RME performers. Statistical significance for t-tests was set at *p* < 0.006 (incorporating a Bonferroni correction for the number of comparisons, *p* = 0.05/8 = 0.006) for clinical scales, at *p* < 0.008 (Bonferroni corrected for the number of comparisons, *p* = 0.05/6 = 0.008) for assessment of memory and language and at *p* < 0.005 (Bonferroni corrected for the number of comparisons, *p* = 0.05/11 = 0.005) for attentional and executive function performance.

The perception of sensations scores was compared between active and sham tDCS in each group using the Wilcoxon matched-pair test.

## 3. Results

Overall, elderly participants achieved lower scores in the RME task than young participants (elderly individuals: Mean: 22.1 SE: 0.8; young individuals: 24.3 SE: 0.9, t(60) = −2.45, *p* = 0.017). The RME task median value was used to define the high and low categories of the performance variable for each of the four groups, so that the median RME value was 23 for elderly female participants, 22 for elderly male participants and 24 for both young female and male participants.

### 3.1. Active tDCS Effects Recorded in High- and Low-Performing Elderly and Young Participants

ANOVA indicated the significant effect of “Group” (F(3,54) = 7.34, *p* = 0.003, η^2^ = 0.29) and “Performance” (F(1,54) = 5.08, *p* = 0.028, η^2^ = 0.09) and of the interaction between “Group” and “Performance” (F(3,54) = 3.18, *p* = 0.031, η^2^ = 0.15). Post hoc analysis showed that only the RME high-performing elderly female group reported smaller active tDCS effects in the AI task than the low-performing elderly female individuals (elderly female participants group = low-performing: 445.0, SE: 103, high-performing: 38.9, SE: 46, *p* = 0.014; elderly male participants group = low-performing: 79.6, SE: 33, high-performing: 0.0, SE: 59, *p* = 0.90; young female participants group = low-performing: −107.2, SE: 86, high-performing: −130.4, SE: 67, *p* = 0.90; young male participants group = low-performing: 62.6, SE: 75, high-performing: 82.8, SE: 75, *p* = 0.90). Elderly female participants also exhibited different active tDCS effects compared to young female (high-performing: *p* = 0.00003, low-performing: *p* = 0.0005) and young male (high-performing: *p* = 0.046, low-performing: *p* = 0.027) groups.

### 3.2. RME Task Score and AI Sham Performance Recorded in High- and Low-Performing Elderly and Young Participants

One-way ANOVA was performed on the RME task score and on AI sham performance (RTs and accuracy) to evaluate for differences among the participant groups. In particular, considering the statistically significant post hoc analysis of the active tDCS effect in elderly females, ANOVA was performed on data from five groups: (1) young female participants group, (2) young male participants group, (3) elderly male participants group and elderly female group split into (4) high- and (5) low-performing participant subgroups.

Regarding the RME task, ANOVA indicated the significant effect of “Group” (F(4,57) = 4.69, *p* = 0.002, η^2^ = 0.25). Post hoc analysis showed that low-performing elderly female participants (20.4, SE: 1.3) reported lower scores than the young female group (25.1, SE: 0.8; elderly low-performing female participants group vs. young female group *p* = 0.04).

**Figure 2:**
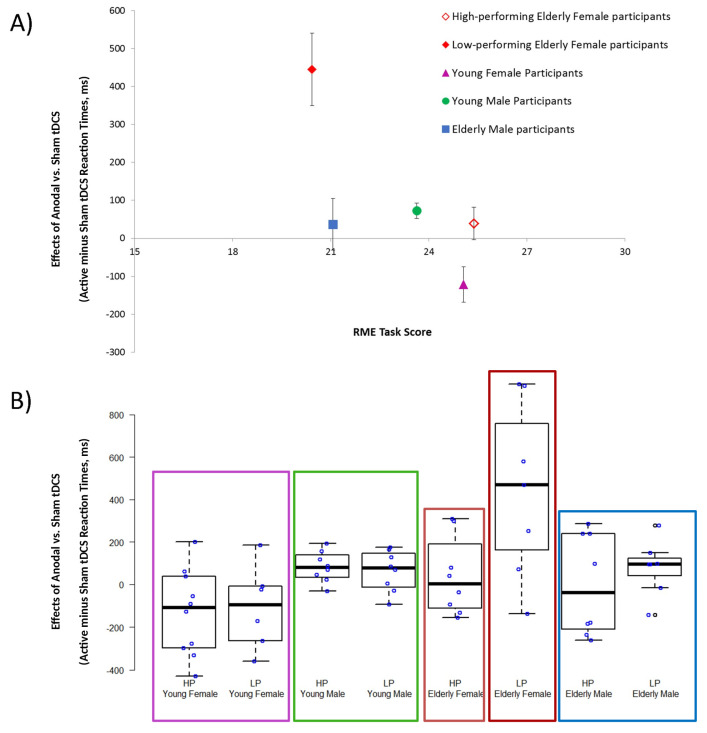
Panel A shows mean active transcranial Direct Current Stimulation (tDCS) effects in the Theory of Mind (ToM) task and the errors bars indicate mean standard errors. Panel B displays the distribution of Reading the Mind in the Eyes (RME) raw scores using boxplots. Blue points represent the single participant raw scores. HP = high-performing; LP = low-performing. The elderly female group was split into high- and low-performing subgroups based on the median value obtained during the RME task and exhibited differences in the active tDCS effects in the ToM task. In particular, higher tDCS effects were evident in elderly female participants with lower RME performances, whereas elderly female participants with higher performances showed lower active tDCS effects. Moreover, a comparison of these two elderly female subgroups with previously reported young female individuals [27] showed that high-performing elderly female individuals had an RME task score similar to that obtained by young female individuals, reporting a trend for a different effect of active tDCS. Otherwise, low-performing elderly female individuals showed a significantly lower RME task score and a different effect from active tDCS compared to young female individuals.

In relation to AI sham performance, ANOVA for accuracy indicated a statistically insignificant effect of “Group” (F(4,57) = 2.02, *p* = 0.11, η^2^ = 0.11), whereas ANOVA for RTs showed the significant effect of “Group” (F(4,57) = 14.50, *p* < 0.001, η^2^ = 0.50). Post hoc analysis showed that young male (1132 ms, SE: 91) and young female (1096 ms, SE: 91) participants obtained faster RTs than elderly male (1704 ms SE: 94) and elderly female (low-performing: 2092 ms, SE: 138; high-performing: 1640 ms, SE: 129) participants (elderly low-performing female participants vs. young female group, *p* < 0.001; elderly high-performing female participants group vs. young female group, *p* < 0.001; elderly male participants vs. young male group, *p* < 0.001), whereas no difference was recorded between high- and low-performing elderly female participants (*p* = 0.21). See Figure 2 for a graphical representation.

### 3.3. Clinical and Neuropsychological Performances Achieved by High- and Low-Performing Elderly Subgroups

To compare the demographic characteristics and the clinical as well as neuropsychological performance achieved by the RME high- and low- performing elderly subgroups, t-tests were used. The results, as shown in Supplementary Table S1, showed that high-performing elderly female participants obtained better scores on an executive functioning task (congruence effect in the Flanker task), on verbal short-term memory test (digit span forward) and language (semantic verbal fluency) abilities than low-performing participants, whereas high-performing elderly male participants performed better on the MMSE and language task (phonemic verbal fluency) than low-performing elderly male individuals.

### 3.4. TDCS-Sensations Questionnaire

For each of the four groups, the tDCS sensations questionnaire scores reported during active tDCS were compared with those reported during the sham tDCS using a Wilcoxon matched-pair test, which showed comparable tDCS-induced sensations in the two stimulation conditions (elderly female participants group = Active: 1.58, SE 0.26, Sham: 1.53, SE 0.3, T = 46.0, z = 0.80, *p* = 0.43; elderly male participants group = Active: 1.03 SE 0.3, Sham: 1.20, SE 0.3, T = 51.5, z = 0.48, *p* = 0.63; young female participants group = Active: 1.50, SE 0.2, Sham: 1.00 SE 0.1, T = 7.0, z = 1.18, *p* = 0.15; young male participants group = Active: 2.0, SE 0.4, Sham: 1.31 SE 0.2, T = 7.0, z = 1.84, *p* = 0.07). Overall, only few subjects reported low-intensity sensations (burning and itching).

## 4. Discussion

Brain aging is characterized by changes in cognitive abilities that may result from neural plasticity, although the functional role of these changes remains unclear [59]. Despite the widespread interest in these changes described during human aging, little is known about the relationship between age and ToM performance [9], and the existing evidence on ToM age-related changes is conflicting [60]. Sex-related differences in ToM changes with aging [61] are also of interest, even though very few studies have demonstrated a specific sex-related advantage on such abilities with aging.

In the present study, in attempt to understand differences related to age, sex and ToM performance, we categorized elderly and young participants (male and female) into ToM high-performing and low-performing groups, based on the RME task score. In agreement with the literature, we found that elderly participants show a significant decline in ToM compared to that in young participants [60].

We subsequently compared the effects of active tDCS applied over mPFC to the AI task in high-performing elderly participants, low-performing elderly participants and high and low-performing young individuals. In elderly female participants, exclusively low-performing individuals experienced slowing of reaction times in the AI task when active tDCS was applied (vs. sham stimulation), as reported in our previous study on elderly individuals [29]. This finding is in line with those of previous studies showing that active tDCS may exhibit differential effects depending on the stimulated area, type of task and timing of stimulation [62]. In particular, recent studies have reported increased reaction times in a facial emotion identification task [63] and greater difficulties in distinguishing between self and other faces [64] following anodal tDCS. It should be noted that in the elderly female low-performing group, the significant slowing of RTs induced by anodal tDCS was accompanied by lower executive control abilities [65] and lower performance on verbal fluency and short-term memory. These data are in line with those of studies on the role of executive functions in ToM performance and highlight the importance, at least for aging, of inhibition and updating processes in working memory for understanding mental states. Some studies have supported the hypothesis that mPFC is actively involved in attentional processing, especially in maintaining attentional capacities, and is involved in the top-down attention to one’s own emotions [66]. This hypothesis is in line with our results demonstrating a link between the significant slowing of RTs induced by active tDCS and lower executive control abilities.

In the present work, active tDCS modulated mPFC in the ToM low-performing (but not high-performing) elderly female participants, suggesting that ToM capacities of those in the high-performing (but not low-performing) group could be controlled by other brain regions such as the posterior regions involved in attentional control processes. To the best of our knowledge, there is no neuroimaging evidence to suggest that ToM high- and low-performing healthy elderly individuals show different brain activation patterns. Instead, some neuroimaging studies that have investigated other cognitive domains (i.e., memory or language abilities) in elderly individuals have suggested that high-performing elderly adults may show different brain activations compared to young and/or low-performing elderly participants [67,68].

Some studies have associated abilities in executive functions to deteriorations in ToM processing during healthy aging [69,70,71]. These data are not surprising because brain regions associated with ToM and executive abilities, such as the dorsolateral prefrontal cortex and the anterior medial frontal cortex, are known to be profoundly impacted by aging [72]. Moreover, it is noteworthy that mPFC is a part of the default mode network, which is associated with different social cognition abilities, similar to hot executive functions that integrate motivational and emotional processes [73]. 

Interestingly, shifts in default mode network responsivity have been described, in particular in sex-specific functional changes during healthy aging [29], with a greater efficiency in posterior default mode network for healthy elderly males and in anterior executive network for healthy elderly females, have been reported [74].

One can speculate about the influence of compensatory mechanisms related to age and sex, for which a good performance in the ToM task requires the involvement of a brain network separate from those active in young women. Alternatively, low-performing participants who exhibit worse executive functioning abilities may show altered activity in cortical areas that could potentially disturb the effect of tDCS on mPFC.

According to the literature on social and affective abilities in aging, which mention that age differences result from different strategies on how resources are allocated in the elderly as compared with the young [69,75,76,77,78,79], our results show a relationship between the significant slowing of RTs induced by active tDCS and the lower abilities in executive control. This evidence may suggest that the current pattern of sex-related differences on ToM ability is related to less efficient strategic processing applied by low-performing elderly female participants during a ToM task.

Neural plasticity allows the neural decline that accompanies physiological aging to be counteracted by means of structures and functions in brain modification [80]. It has been proposed that the implication of this process is the recruitment of different “neural strategies” to maintain the cognitive capability [81,82]. Different hypotheses have repeatedly attempted to explain the functional changes recorded in the elderly [83,84,85]. Age-related neural changes have been dynamically accounted by the scaffolding theory of aging and cognition (STAC; [86]). Accordingly, the age-related neural changes stimulate the compensatory scaffolding or support mechanisms which helps maintain a high level of cognitive functioning. This process, in accordance with previous neuroimaging studies, would result in evidence of overactivation or reduced lateralization of different brain areas, such as the frontal cortex and the parietal, medio-temporal and occipital regions [81,85,87,88,89,90,91].

The brain dysfunction hypothesis accounts for neural changes during aging as evidence of a low functioning brain [92,93,94,95,96]. New evidence from functional neuroimaging has shown the activation of similar brain areas in both young and elderly participants with high cognitive performance [67,68,97,98].

Overall, our data showed slower reaction times in the AI task with active tDCS in elderly female low-performing participants, which are in line with the brain dysfunction hypothesis. In fact, low-performing elderly female individuals who showed significant slowing of RTs induced by active tDCS also had lower scores in tests evaluating executive functions, verbal fluency and verbal short-term memory.

Our data do not confirm the canonical assumption of improved performance induced by anodal tDCS; this finding is in line with those reported in previous studies showing that anodal tDCS may exhibit differential effects during cognitive tasks [63]. Our results may depend on the stimulated area, type of task and timing of stimulation [21,62,99]. The mechanisms underlying the effects of tDCS are not yet entirely understood, but they could involve changes in the synaptic microenvironment by modifying synaptic strength or GABA-mediated activity that modifies the densities of ion channels localized in the cortical area below the electrodes, which would interfere with brain excitability [19,62,100,101,102]. Moreover, extensive literature has shown that brain stimulation induces highly variable effects across individuals [62] and can affect females and males differently [25,27,103].

There are limitations to our study. First, given that the sample size included in this pilot study was relatively small, the findings reported here need to be reproduced in larger cohorts before drawing firm conclusions. Second, as we did not vary the stimulation target area, we cannot confirm the specificity of the mPFC for the effects observed. Moreover, lack of a complete neuropsychological assessment in the younger participants did not allow for a direct assessment of the influence of executive functioning on ToM performance in this population. Finally, slightly different stimulation intensities have been used in young and elderly participants and, accordingly, some of the different effects recorded in the two age groups could be linked to these methodological differences. 

Further studies using a combination of non-invasive brain stimulation techniques and neuroimaging approaches (e.g., electroencephalography, functional near-infrared spectroscopy and functional magnetic resonance imaging) could establish a more comprehensive examination of both local and global neuroplastic changes. In particular, multimodal approaches combining transcranial electrical stimulation and other neuroimaging and/or neurophysiology techniques (e.g., transcranial magnetic stimulation—electroencephalography/electromyography) could lead to a deeper understanding of the effects and neurological mechanisms of tDCS [104].

In conclusion, our data suggest a relationship between age, sex and ToM, but further studies are needed to confirm this. Nevertheless, our results strengthen those reported in previous studies that describe sex-related differences in social cognition function. Through the use of non-invasive brain stimulation, we have provided further evidence showing slowing of ToM responses during active tDCS over mPFC relative to sham, which was previously recorded exclusively in elderly females [29]. Using tDCS, we found a possible role of mPFC specifically in elderly ToM low-performing females in comparison to high-performing elderly females, who obtain ToM performances similar to young female participants. Taken together, we suggest that the use of a new functional strategy could contribute to maintaining ToM performance during aging. This is observed, in low-performing elderly female participants, who perform worse than high-performing elderly female and young female participants, arguably because they use a less efficacious strategy. Moreover, low-performing elderly female participants exhibit a decline in executive functioning compared to high-performing elderly female individuals. Variation in ToM performance can at least partly be explained by differences in the functional areas recruited in young and elderly participants.

## Supplementary Materials

The following are available online at https://www.mdpi.com/2076-3425/10/5/257/s1, Table S1: Demographical, clinical and neuropsychological data of elderly groups.
