# Supplementary material for: Theory of Mind Performance Predicts tDCS-Mediated Effects on the Medial Prefrontal Cortex: A Pilot Study to Investigate the Role of Sex and Age
Source: Brain Sci. 2020 Apr 28;10(5):257. doi: 10.3390/brainsci10050257 (PMC7288024; doi:10.3390/brainsci10050257)
Supplement: Supplementary file 1 [file brainsci-10-00257-s001.pdf]

Table S1. Demographical, clinical and neuropsychological data of elderly groups.

	Male Elderly Participants				Female Elderly Participants				
	**Total (n=15)**	**HP (n=8)**	**LP (n=7)**	**p-value**	**Total (n=15)**	**HP (n=8)**	**LP (n=7)**	**p-value**	**Cut-off**
Age (years)	68.3(5.0)	69.3(4.5)	67.3(5.4)	0.80	67.5(7.1)	65.4(5.1)	70.0(3.8)	0.75	
Education (years)	10.4(4.6)	13.6(4.5)	6.7(2.5)	0.06	11.6(4.3)	13.1(3.8)	9.9(4.5)	0.15	
Interpersonal Reactivity Index (IRI), total score	88.5(7.6)	92.0(7.5)	84.6(8.2)	0.23	90.0(8.5)	89.1(7.2)	91.0(8.9)	0.70	
Reading the Mind in the Eyes Test (RME)	21.1(4.8)	24.5(2.3)	17.1(3.1)	**0.0001**	23.1(3.1)	25.4(2.3)	20.4(4.7)	**0.0001**	
**Mood and Anxiety Assessment**									
Geriatric Depression Scale (GDS)	2.4(2.2)	2.0(2.0)	2.9(2.1)	0.35	4.8(4.6)	4.9(5.1)	4.7(1.8)	0.95	<11
State-Trait Anxiety Inventory (STAI)-State	30.4(7.0)	28.3(2.9)	32.9(9.9)	0.33	31.9(6.7)	32.4(7.5)	31.3(6.4)	0.77	
State-Trait Anxiety Inventory (STAI)-Trait	33.9(6.9)	32.8(4.3)	35.3(9.0)	0.35	36.2(8.8)	38.5(9.1)	33.6(6.4)	0.31	
**Subjective Memory Complaints**									
Everyday Memory Questionnaire (EMQ)	46.1(15.7)	42.6(11.5)	50.0(18.7)	0.53	45.5(15.2)	44.9(20.0)	46.3(10.7)	0.87	
**Cognitive Reserve**									
Cognitive Reserve Index (CRI – q), total score	111.3(15.9)	121.3(12.8)	100.0(14.6)	0.05	116.4(15.6)	117.6(17.2)	115.0(16.2)	0.77	
**Cognitive Assessment**									
**Screening for dementia**									
MMSE	**28.9(0.8)**	**29.5(0.5)**	**28.3(0.7)**	**0.003**	28.9(0.9)	29.4(0.7)	28.4(1.3)	0.05	≥24
**Non-Verbal Reasoning**									
Raven's colored progressive matrices	30.8(3.3)	33.0(2.0)	28.3(3.5)	0.03	29.7(4.7)	30.9(4.0)	28.3(3.5)	0.32	>17.5
**Memory**									
Digit Span (forward)	5.6(1.1)	5.8(0.5)	5.4(1.5)	0.73	**6.0(1.4)**	**6.9(1.1)**	**5.0(0.8)**	**0.004**	**>4.25**
Digit Span (backward)	4.6(1.1)	5.0(0.9)	4.1(1.2)	0.10	4.7(1.0)	5.3(0.7)	4.0(1.1)	0.01	>2.64
Story Recall	12.5(3.4)	13.2(4.0)	11.8(3.3)	0.39	14.0(3.8)	15.8(4.2)	12.0(3.7)	0.06	>7.5
RAVLT (Immediate recall)	44.0(8.6)	46.9(8.5)	40.7(8.5)	0.27	47.6(7.5)	50.9(7.2)	43.9(9.6)	0.08	>28.52
RAVLT (Delayed recall)	8.5(2.3)	9.3(3.1)	7.7(1.9)	0.39	10.2(2.8)	11.3(2.5)	9.0(2.2)	0.14	>4.68
Rey-Osterrieth Complex Figure, recall	17.8(5.6)	20.1(6.6)	15.2(4.7)	0.19	16.6(5.9)	19.1(6.2)	13.8(5.8)	0.09	>9.46
**Language**									
Token Test	33.7(1.5)	34.6(1.1)	32.6(1.6)	0.04	34.0(1.5)	34.3(1.3)	33.7(1.6)	0.54	>26.25
**Verbal Fluency, phonemic**	**36.0(9.1)**	**40.9(7.1)**	**30.4(7.3)**	**0.003**	39.1(13.7)	45.0(15.6)	32.4(9.7)	0.09	>16
Verbal Fluency, semantic	49.1(8.8)	54.3(8.2)	43.1(7.9)	0.06	**48.9(11.6)**	**56.9(9.3)**	**39.7(7.5)**	**0.001**	**>24**
Naming Objects (B.A.D.A)	28.9(0.8)	29.4(0.5)	28.3(0.7)	0.009	27.9(2.2)	28.6(1.1)	27.1(1.0)	0.22	
Naming Actions (B.A.D.A)	26.5(1.0)	26.8(0.8)	26.1(1.0)	0.35	25.5(2.6)	26.5(0.9)	24.3(1.5)	0.11	
Wechsler Adult Intelligence Scale – Vocabulary	38.6(13.2)	47.1(6.7)	28.9(15.5)	0.05	44.0(7.5)	47.0(4.8)	40.6(12.8)	0.11	
**Praxis**									
Rey-Osterrieth Complex Figure (ROCF), copy	31.6(2.8)	32.0(3.2)	31.1(2.7)	0.63	30.9(4.2)	32.8(1.9)	28.8(2.3)	0.08	>28.87
**Attentional and Executive Functions**									
Trial Making Test-A (seconds)	35.9(6.9)	33.3(5.2)	38.9(8.0)	0.09	41.5(14.7)	38.8(15.1)	44.7(7.7)	0.47	<94
Trial Making Test-B (seconds)	137.2(65.0)	107.1(29.0)	171.6(86.9)	0.17	118.8(59.6)	84.6(13.5)	157.9(76.7)	0.02	<283

Stroop test – interference effect on time (seconds)	24.5(11.9)	19.6(2.8)	30.1(14.6)	0.03	23.9(8.2)	20.1(4.2)	28.2(13.1)	0.06	<36.92
Stroop test – interference effect on errors	0.6(1.0)	0.4(0.5)	0.9(1.3)	0.22	0.8(1.0)	0.5(0.8)	1.1(1.0)	0.26	<4.24
Wisconsin Card Sorting Test (WSCT) – Global score	66.5(35.6)	56.8(31.0)	77.6(34.7)	0.06	58.5(42.6)	46.1(38.6)	72.6(34.5)	0.26	<90.6
Wisconsin Card Sorting Test (WSCT) – Perseverative responses	21.4(18.9)	15.0(8.4)	28.7(18.6)	0.10	22.2(17.0)	18.8(17.7)	26.1(16.8)	0.44	<42.7
Wisconsin Card Sorting Test (WSCT) – Non Perseverative errors	20.9(12.1)	20.8(11.5)	21.1(13.7)	0.28	17.1(13.2)	11.8(9.6)	23.1(11.8)	0.11	<30.0
Wisconsin Card Sorting Test (WSCT) – Failure to maintain the set	1.5(1.2)	1.3(1.0)	1.9(1.2)	0.11	1.0(1.1)	1.1(1.1)	0.9(1.1)	0.66	<4.0
Flanker Task – Effect of Incongruency on RTs (milliseconds)	+174.4(71.8)	+195.9(81.4)	+149.7(56.0)	0.06	+134.1(98.7)	+80.8(94.5)	+195.0(70.6)	0.02	
Flanker Task – Effect of Congruency on RTs (milliseconds)	+2.2(51.9)	-15.0(39.4)	+21.8(65.8)	0.30	**+0.5(45.5)**	**-29.7(43.7)**	**+35.0(55.9**	**0.002**	

Raw scores are reported (SD between parentheses). HP: high-performing participants to the Reading the Mind in the Eyes Test; LP: low-performing participants to the Reading the Mind in the Eyes Test; MMSE: Mini Mental State Examination, RAVLT: Rey Auditory Verbal Learning Test, B.A.D.A (Batteria per l'Analisi dei Deficit Afasici), RTs: Reaction Times. *p-value related to the t-tests comparing high- and low-performing subgroups into each experimental elderly group. Values in bold indicate significant difference (Bonferroni corrected for the number of comparisons: p<0.006 for clinical scales, p < 0.008 for memory and language and p < 0.005 for attentional and executive functions evaluations). Cut-off scores according to Italian normative data are reported.
